# Large Scale Explorative Oligonucleotide Probe Selection for Thousands of Genetic Groups on a Computing Grid: Application to Phylogenetic Probe Design Using a Curated Small Subunit Ribosomal RNA Gene Database

**DOI:** 10.1155/2014/350487

**Published:** 2014-01-06

**Authors:** Faouzi Jaziri, Eric Peyretaillade, Mohieddine Missaoui, Nicolas Parisot, Sébastien Cipière, Jérémie Denonfoux, Antoine Mahul, Pierre Peyret, David R. C. Hill

**Affiliations:** ^1^UMR CNRS 6158, ISIMA/LIMOS, Clermont Université et Université Blaise Pascal, F63173 Aubière, France; ^2^Clermont Université et Université d'Auvergne, EA 4678 CIDAM, BP 10448, F63001 Clermont-Ferrand Cedex 1, France; ^3^Clermont Université et Université d'Auvergne, UFR Pharmacie, F63001 Clermont-Ferrand Cedex 1, France; ^4^CNRS, UMR 6023, LMGE, F63171 Aubière, France; ^5^Clermont Université, CRRI, F63177 Aubière, France

## Abstract

Phylogenetic Oligonucleotide Arrays (POAs) were recently adapted for studying the huge microbial communities in a flexible and easy-to-use way. POA coupled with the use of explorative probes to detect the unknown part is now one of the most powerful approaches for a better understanding of microbial community functioning. However, the selection of probes remains a very difficult task. The rapid growth of environmental databases has led to an exponential increase of data to be managed for an efficient design. Consequently, the use of high performance computing facilities is mandatory. In this paper, we present an efficient parallelization method to select known and explorative oligonucleotide probes at large scale using computing grids. We implemented a software that generates and monitors thousands of jobs over the European Computing Grid Infrastructure (EGI). We also developed a new algorithm for the construction of a high-quality curated phylogenetic database to avoid erroneous design due to bad sequence affiliation. We present here the performance and statistics of our method on real biological datasets based on a phylogenetic prokaryotic database at the genus level and a complete design of about 20,000 probes for 2,069 genera of prokaryotes.

## 1. Introduction

The total number of species on our planet is of about 9 million, according to the latest biodiversity estimate. However, the vast majority of these species are not yet discovered and only over 1.2 million species have been already catalogued in a central database [1]. Most nondescribed species are microorganisms. Microbial communities represent the most important and diverse group of organisms living on earth. They play an important role in the functioning of ecosystems [2]. The comprehension of the role of microorganisms is then a major challenge of microbial ecology. Because of the huge microbial biocomplexity, high-throughput molecular tools allowing simultaneous analyses of existing populations are well adapted to survey microorganisms in complex environments [3].

Phylogenetic Oligonucleotide Arrays (POAs) are currently widely used and are one of the most promising approaches for studying microbial communities. They generally use oligonucleotide probes to target small subunit ribosomal RNA (SSU rRNA) genes and discriminate organisms. SSU rRNA gene is a phylogenetic biomarker largely used in the majority of studies. However, the sequences could be highly conserved leading to some difficulties for species discrimination. Consequently, specific oligonucleotide probes selection for POAs could be a very difficult task to obtain a high resolution level [4].

Efficient oligonucleotide probes must have the following two properties: sensitive and specific. The sensitivity of a probe means its capacity to detect low levels of its complementary target in complex samples. A sensitive probe is one that is able to access its complementary sequence in the target and returns a strong signal when the target is present in the hybridized sample. The sensitivity generally increases with probe length as the binding energy for longer probe/target hybrid complexes is typically higher and hybridization kinetics are irreversible.

The specificity of a probe means its capacity to hybridize only with its complementary counterpart target. A specific probe is one that does not cross-hybridize with a nontarget sequence and returns a weak signal when the target is absent from the hybridized sample. The specificity generally decreases with the increase of probe length: short oligonucleotide probes are more specific, allowing discrimination of single nucleotide polymorphisms under optimal conditions, but at the cost of reduced sensitivity. The specificity is the most important criterion of the probes quality measure in probe design algorithms [5]. Probe design algorithms usually use specific algorithms such as suffix array method or BLAST [6] to check the specificity of probes by searching possible cross-hybridizations against datasets. However, the exponential increase of the number of sequences deposited in public databases induced an important increase in the computational capacity requirements of oligonucleotide probe design algorithms [7] and also a fundamental change in the way these algorithms are designed.

It is true that we can find fast probe design software running on regular PCs because they allow selecting probes for few DNA sequences or/and do not check the specificity of the obtained probes. The probe specificity tests against the large and ever growing biological datasets require a particular attention to develop a new generation of probe design software able to deal with high performance computing. In this context, parallel and distributed architectures such as computing clusters or computing grids [8] can provide interesting performances. Computing grids provide a promising approach to use distributed resources to meet the continuously evolving computational needs of bioinformatics tools [9]. They are particularly suited when the parallelism can be based on data splitting providing true independent computing [10]. They allow a transparent use of geographically dispersed resources for largescale distributed applications. They are adapted for time consuming algorithms that can be split into several independent jobs.

In addition to the use of known probes in POAs that allow us to simultaneously study several thousand known organisms, it is also important to design explorative probes that can detect unknown sequences not yet available in public databases and explore the vast majority of microorganisms that are still nondiscovered [3].

Here, we present a new parallelization method of a probe design algorithm to select known and explorative oligonucleotide probes using a computing grid. This software runs on the European Grid Infrastructure (EGI). EGI is a multidisciplinary grid infrastructure providing more than 250.000 CPU cores and more than 100 petabytes over 51 countries (http://www.egi.eu/). We introduced an efficient parallelization method to take advantage of the computing power available in the EGI grid to perform largescale oligonucleotides selection. We present also a new algorithm for the construction of a personal high-quality phylogenetic database that can be used to select specific, sensitive, and explorative probes targeting any prokaryotic or fungal taxonomic group, for phylogenetic oligonucleotide microarrays.

## 2. Related Works and Limitations

Phylogenetic Oligonucleotide Arrays (POAs), targeting the SSU rRNA genes, are known as one of the most interesting approaches to study the microbial diversity in complex environments [11]. In the last ten years, several works were done to study the biodiversity of different environments using such POAs. A microarray composed of 132 probes of length 18 mers was proposed to monitor prokaryotic microorganisms involved in sulphate reduction [12]. Another microarray considered as the most evolved POA called “PhyloChip” was developed by Brodie et al. [13] based on the Affymetrix GeneChip platform. The PhyloChip is composed of nearly 500 000 oligonucleotide probes targeting almost 9000 operational taxonomic units. This tool has been used to characterize prokaryotic communities from various ecosystems [13–17].

Additionally, several tools were proposed to select probes for phylogenetic arrays; they are discussed hereafter and in Dugat-Bony et al. [3].

The PRIMROSE program [18] was proposed to select both oligonucleotide probes and PCR primers. The probe design mechanism of PRIMROSE consists in first producing a multiple alignment for a given group of sequences. Probes are then selected and subsequently tested against an input database, to search for potential cross-hybridizations and to verify the coverage of the targeted group of sequences. PRIMROSE has been mainly used in PCR-based and FISH (fluorescent in situ hybridization) approaches [19, 20], but only a few applications of POAs using PRIMROSE have been reported [21]. The PRIMROSE tool does not allow selecting explorative probes. The ARB software package [22] proposed a probe design tool that allows selecting oligonucleotide probes with a length of 10 to 100 mers. This tool consists in searching all possible signature sequences of a targeted group of organisms specified by the user. Probes are then selected and matched against a database using the ARB Probe Match software. The ARB probe design tool has been used to design low-density custom-made POAs, composed of only a few hundreds of probes [23–25]. However, this probe design software is not well suited for large scale oligonucleotide probe design. Furthermore, it allows selecting only probes targeting known organisms and does not allow selecting explorative probes.

ARB and PRIMROSE tools allow selecting promising probes or primers for a single organism or a group of related organisms. However, emerging experimental approaches seek to simultaneously detect numerous organisms of interest thereby requiring the identification of large numbers of compatible probes [7, 26].

Oligonucleotide retrieving for molecular applications (ORMA) [27] is one of the most recent software proposed to select oligonucleotide probes. ORMA is composed of a set of scripts developed under Matlab and uses the BLAST program to check the specificity of the oligonucleotide probes selected. It allows designing probes for molecular application experiments on sets of highly similar sequences. ORMA was first applied to the design of probes targeting 16S rRNA genes, but it can also be used on any set of highly correlated sequences. This probe design tool has been used to design the HTF-Microbi-Array allowing high taxonomic level fingerprinting of the human intestinal microbial community [28].

All of these programs allow selecting probes targeting only known microbial communities with available sequences in public environmental databases. A few tools such as PhylArray [29] were designed with the possibility of selecting explorative probes for phylogenetic microarrays. PhylArray was developed with the Perl language. It allows selecting probes for a group of SSU rRNA sequences to globally survey known and unknown bacterial communities. Probe selection using PhylArray can take several days for only one large group of sequences.

In this work, we present a new parallel approach to select both known and explorative oligonucleotide probes on computing grids. The probe design strategy is based on the original algorithm PhylArray described in Militon et al. [29].

## 3. Material and Methods

### 3.1. Implementation

Our method was implemented in a program called PhylGrid 2.0. It was developed under Linux CentOS 5.4 with C++ and Perl. It uses three other programs: BLAST [6], Clustalw-MPI [30], and Opal [31].

Our approach hides the EGI grid to the user who just uses a regular computer which acts as a grid UI (User Interface: a grid component for user access to the grid). The first step was to implement the software on the User Interface (UI). This allows a direct connection to the EGI grid using a proxy authentication for the submission of multiple jobs. The main resources used by our grid application are the Workload Management System (WMS), a Berkeley Database Information Index (BDII), Computing Elements (CEs), and Storage Elements (SEs). We used the gLite middleware API commands. Submission, jobs management, and file transfer were implemented.

### 3.2. SSU rRNA Database Building

Probe design requires building a SSU rRNA database used as input and also as a reference database to check the specificity of all possible probes. This database must be of high quality in order to obtain the right design and to avoid wrong cross-hybridization results caused by poor sequences quality and erroneous affiliation in public environmental databases. Here, we developed a new algorithm to revisit, for more precision, the initial database described in Militon et al. [29].

All SSU sequences of the taxonomic divisions Prokaryotes (PRO), fungi (FUN), and environmental samples (ENV) downloaded from the European Molecular Biology Laboratory (EMBL) nucleotide sequence database were used as a reference to build our database carefully crafted for our probe design software. Several steps were needed. First, small subunit rRNA gene sequences (16S for prokaryotes and 18S for fungi) were extracted and filtered according to their quality and size. We kept only the sequences that met the following criteria.

- The sequence length is greater than 1,200 bases.
- The sequence length is smaller than 1,600 bases for prokaryotic sequences and 1,800 bases for fungal sequences.
- The sequence is assigned to the genus level in EMBL database (taxonomic information is extracted from the (OC) organism classification EMBL field).
- The percentage of unknown nucleotides (not {A, C, G, T}) in the sequence is less than 1%.
- The maximum number of consecutive unknown bases must not exceed 5. The last two criteria allow removing low quality sequences.

These stringent parameters were chosen in order to allow an efficient probe design. Then, extracted sequences were grouped at the genus taxonomic rank and each group was included in its specific kingdom (prokaryote or fungi) according to the NCBI taxonomy database.

The next step consists in checking the orientation of the obtained sequences. We used BLASTN program and a reference sequence to check and correct the orientation of sequences that had been incorrectly oriented in the EMBL database.

Subsequently, a BlastClust was made on each group to eliminate redundant sequences, using the following parameters allowing a single-linkage clustering at 100% identity cut-off:

-p F (nucleotide sequences);
-S 100 (similarity threshold);
-L 1 (minimum length coverage);
-b F (required coverage as specified by -L and -S on only one sequence of a pair).

Finally, for each group, we checked the homogeneity of its sequences. The aim was to eliminate sequences badly annotated and to create a homogeneous group of sequences to allow selecting specific probes for this group. This step was done using a modified version of Clustalw [32] to compute distances between sequences and the K-means method [33] to clustering sequences.

We used this algorithm to build a 16S rRNA database at the genus level. We obtained 2,069 prokaryotic genera; each is composed of a set of homogeneous sequences representing the whole diversity. Our algorithm can be easily adapted and used to build high-quality SSU rRNA databases for different taxonomic ranks (family, order, class, etc.).

### 3.3. The Probe Design Algorithm

**Figure 1:**
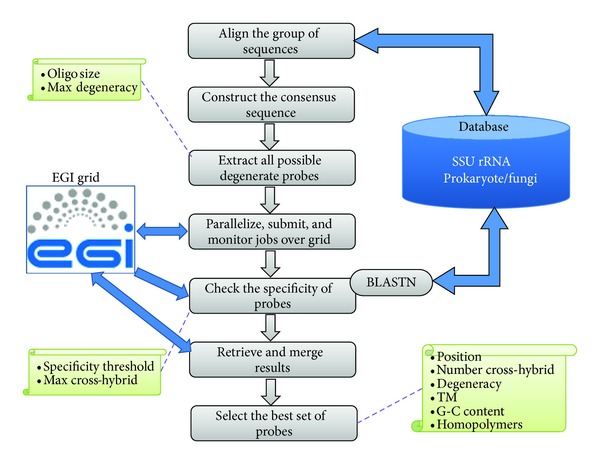
Summary of algorithm steps.

Our algorithm uses 4 main input parameters: probe length, maximum degeneracy of a consensus probe, specificity threshold (the minimum value used to determine if the probe may hybridize with a nontarget sequence), and maximum number of cross-hybridizations.  Figure 1 shows the different steps of our algorithm linked to the EGI grid.

To design probes for an input group of sequences selected by the user, a multiple sequence alignment is first made. For small groups of sequences, Clustalw-MPI [30] is used to align the sequences of the given group. However, for large groups of sequences, the multiple alignment is made in three steps to improve its quality and speed. First, BlastClust is made on each large group (using the parameters -L .98, -S 98, -p F, and -b F) to construct main subgroups of highly similar sequences. Then, sequences of each subgroup are aligned using Clustalw-MPI. Finally, Opal [31] is used to merge the obtained alignments and to reconstitute a complete alignment for the whole group.

The alignment file created is then used to construct a consensus sequence using the IUPAC degenerate nucleotide codes [34]. The aim is not only to obtain a common sequence that entirely represents the whole group of sequences targeted but also to improve alignment and correct possible sequencing errors. For example, in each column of the alignment representing a molecular site, if the number of unknown nucleotides (“N” or gap “-”) is less than half the number of sequences aligned, all the unknown bases of the aligned sequences, at this position, are replaced by the specific or degenerate base calculated from all the specific nucleotides of this position. Else a gap “-” is inserted in the consensus sequence at this position.

The next step of the probe design strategy consists in incrementing a window of length “*l*” (*l* is the length of probes set by the user) along the consensus sequence to find all possible degenerate probes that do not contain gaps (“-”) and whose degeneracy does not exceed the threshold value of maximum degeneracy allowed.

Subsequently, a parallelization is made to distribute all the extracted degenerate probes into “*N*” jobs (*N* is the number of jobs set by the user). For each job, all the degenerate probes are processed. Otherwise, all possible specific and explorative oligonucleotide probes are generated from each degenerate probe, using IUPAC codes [34]. These oligonucleotides are checked for cross-hybridizations against the reference SSU rRNA database initially built, using BLASTN program with the following parameters: “-W 7 -F F -S 1 -e 100 -b 20000”.

Finally, all the obtained regular and explorative oligonucleotide probes are regrouped and saved in a final result file. For each degenerate or specific probe, all the associated information is provided, such as position, degeneracy, number and list of cross-hybridizations, and mismatch positions.

### 3.4. Parallelization Method

Selecting probes for a group of nucleic acid sequences and checking the specificity of each possible probe against a complete SSU rRNA database require a very important computation time. Our software allows running this kind of design on a computing grid. First, the user must choose the number of jobs to use. The consensus sequence, constructed from the alignment file of each group of sequences, is read to extract all possible degenerate probes that do not contain gaps (“-”), based on the probe length set by the user. The degeneracy of each degenerate probe is calculated. If this degeneracy is less than “maximum degeneracy authorized by the user” (MaxDeg), the degenerate probe is saved. A weight value is calculated for each saved degenerate probe based on its degeneracy.

**Figure 2:**
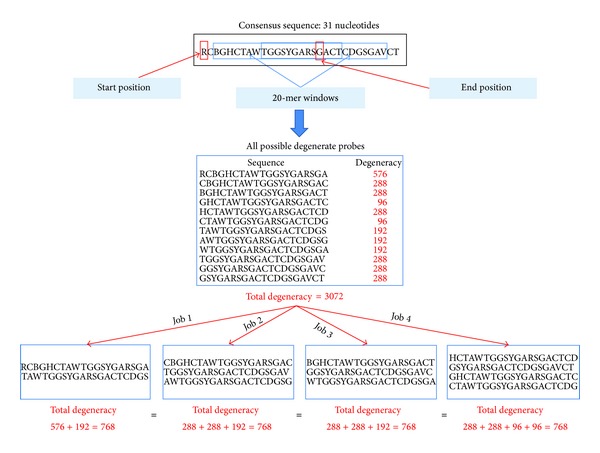
Parallelization strategy to define and submit jobs over the grid.

Once this step is performed, all valid degenerate probes saved are collected and put in the same file. This file must then be cut into “*N*” subfiles (*N* is the number of jobs set by the user) depending on the weight value of each degenerate probe and the sum of all the weight values. First, all the degenerate probes are sorted in descending order based on their weights. The mean degeneracy per subfile is then calculated based on the sum of all the weight values and the number of jobs desired. Finally, a “worst fit” algorithm [35] is used to put each degenerate probe in the largest possible free block in which this degenerate probe can be saved according to its weight. This method allows avoiding the creation of small unusable blocks by making the remainder as large as possible with the aim of making this remainder able to contain other degenerate probes. The subfiles created will have almost the same weight (Figure 2) and the same number of potential probes. Each subfile represents a job that will be submitted to the EGI computing grid.

Moreover, we have developed job monitoring scripts, with resubmission in case of failure to improve the reliability of our grid software. Three cases can be distinguished.

- The job submission failed: the job is resubmitted when a network route is found.
- The job is submitted successfully and failed when executed: a new job is created and submitted.
- The job is submitted successfully and done successfully but the other jobs are not finished: the program waits for all jobs and then merges all results in a single output file.

For running conditions, the database is copied on grid Storage Elements (SEs) accessible to all the grid jobs of a probe design. Regarding submission time, it is important not to overload the Workload Management System (WMS). Otherwise, the program may wait until each job is entirely associated with a CE of the EGI grid before submitting the next job. The following elementary configuration files are necessary to submit jobs successfully on the EGI grid.

- JDL files: each job needs a job description language (JDL) file to be submitted on the Grid.
- Script files: such files describe the elementary tasks that will be executed on the grid. The scripts contain operating system commands and Perl scripts called to perform probe design among all extracted degenerate probes. During execution, SSU rRNA database and subfiles containing degenerate probes are copied on the CE in which the job is running, and Blastn analysis is launched to test cross-hybridization.

Finally, the program is designed to be extensible by separating independently jobs in distinct designs. It creates a single data identifier for each probe design.

## 4. Results

In this section, we present the results obtained by our software on real data sets. We show the performance of our parallelization method compared to the original program PhylArray [29].

### 4.1. Database Building

We developed a new algorithm for the construction of a high-quality curated phylogenetic database, as described above. Our algorithm can be easily adapted and used to build high-quality SSU rRNA databases for different taxonomic ranks (genus, family, order, class, etc.). We used this algorithm to build a SSU rRNA database at the genus level. We obtained about 66,000 16S rRNA gene sequences representing 2,069 prokaryotic genera; each is composed of a set of homogeneous sequences representing the whole diversity. We used PhylGrid 2.0 and this database to create a complete phylogenetic oligonucleotide database composed of about 20,000 probes targeting 2,069 prokaryotic genera.

### 4.2. Alignment of Alignments

Dealing with the multiple sequence alignment for large groups of sequences, an alignment of alignments is achieved to improve the quality and speed of alignment. The alignment time is given in Table 1 for different groups with a varying number of sequences.

For instance, the performance of this method is 4 times faster than a simple multiple alignment when aligning the bacteria genus group “*Bacillus*.”

### 4.3. Load Balancing Method

To distribute the probe design task on all used jobs equitably, we developed a load balancing method based on the degeneracy of all possible degenerate probes extracted from the consensus sequence constructed. To test the efficiency of our method, we compared it to the load balancing method used in the original algorithm PhylArray [29] that selects probes on a computing cluster. To distribute the computation on *N* processors, PhylArray splits the consensus sequence into *N* equal parts. Each part is then processed on a processor.

**Figure 3:**
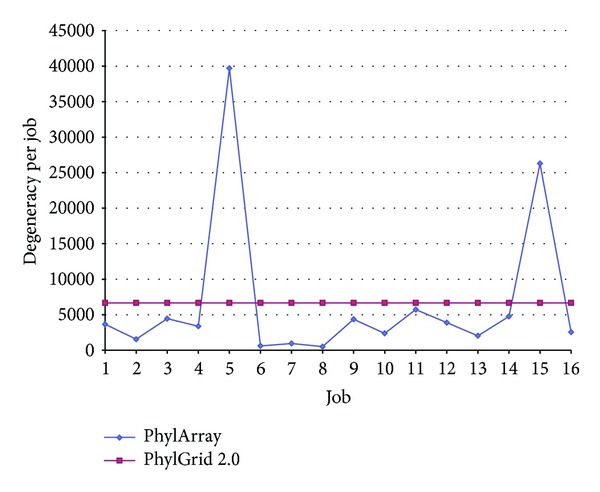
A comparison of our load balancing method with PhylArray [29] using 16 processors to select probes for “*Citrobacter*” group.

**Figure 4:**
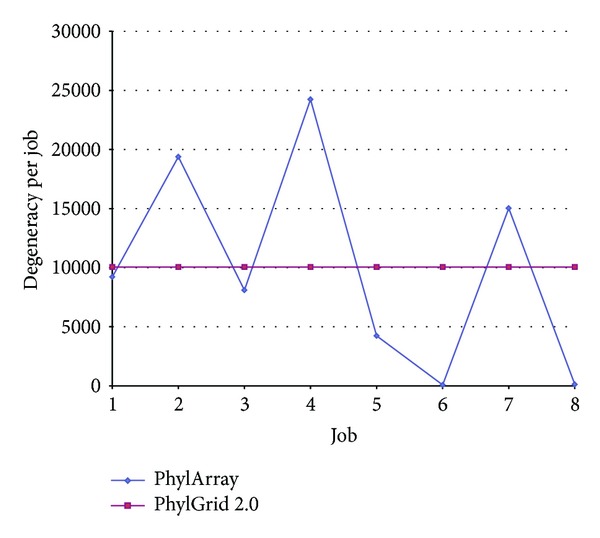
A comparison of our load balancing method with PhylArray [29] using 8 processors to select probes for “*Haemophilus*” group.

The comparison tests were made on real data sets, using respectively 16 jobs to select probes for the genus group “*Citrobacter*” (Figure 3), 8 jobs to select jobs for the genus group “*Haemophilus*” (Figure 4), and finally using 4 jobs to select jobs for 3 genus groups: “*Citrobacter*,” “*Eubacterium,*” and “*Haemophilus*” (Table 2). This comparison shows that our method is more efficient than PhylArray. Using our method the different parts of the probe design, which processed each one on a processor, have almost the same value of degeneracy that is very close to the value of the mean degeneracy per job. For instance, as showed in Table 2, the load standard deviation between jobs is very small (0.5 probe) when using PhylGrid 2.0 compared to the high standard deviation obtained when using PhylArray (18,647.85 probes).

### 4.4. Use of the European Grid EGI

Our software allows users to submit parallel jobs to the EGI computing grid from Biomed Virtual Organization for the purpose of designing probes. To test the performance of our approach, we launched probes design for 10 prokaryotic genus groups simultaneously (“*Eubacterium*,” “*Citrobacter*,” “*Propionibacterium*,” “*Neisseria*,” “*Campylobacter*,” “*Arcanobacterium*,” “*Haemophilus*,” “*Kaistobacter*,” “*Bacteriovorax*,” and “*Riemerella*”), using the following parameters:

- probe length = 25;
- specificity threshold = 0.88 (the probe must not have a similarity greater than or equal to 88%, with a nontargeted sequence);
- maximum number of cross-hybridizations = 100;
- maximum degeneracy = 2000.

**Figure 5:**
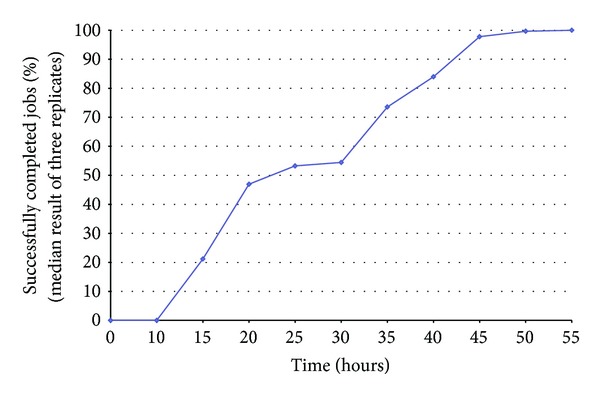
The median execution result of probe selection for 10 genus groups on the EGI grid using 586 jobs.

This task needs more than 8 months to be processed on a single CPU core. We have launched probe designs for these groups on the EGI grid using a total of 586 jobs. We have repeated this test 3 times and the median result in terms of computational time was considered. Finally, we obtained all results successfully after less than 55 hours (with submission and waiting latency). Results are illustrated in Figure 5.

The obtained performance is here of about 106x for 586 jobs despite the submission and waiting latency of the EGI grid. Jobs submitted to a grid spend hours waiting in queues. The unavailability of some grid resources such as a Computing Element or a Storage Element can also cause the loss or blockage of jobs. This can of course increase the global computing time of our software which will however resubmit failed and lost jobs. For instance, in Figure 5, we can see a small decrease in throughput of returned completed jobs in the time window between 20 and 30 hours. This is due to the important resubmission of failed jobs at this computing phase. These jobs were submitted successfully at the beginning, but they failed or were blocked when executed.

## 5. Conclusions

In this work, we show that it is possible to select probes at large scale on a grid infrastructure with significant performance gains, without any particular grid submission optimizations (such as using pilot jobs). Our software allows selecting both specific and explorative (discovery of possible new species) probes with respect to excellent sensitivity and specificity. It takes advantage of the computing power offered by the EGI grid to propose at once probe design for thousands of groups. We also developed job monitoring scripts to improve the reliability and efficiency of our grid software.

The design of oligonucleotide probe on a computing grid requires optimizing the distribution of the probe design algorithm. This is why we developed an efficient parallelization method based on the degeneracy of all possible degenerate probes extracted from the consensus sequence that represents the input group. The probe selection is equally distributed over a given number of jobs. We have compared our parallelization method with the original algorithm PhylArray [29]. We have shown that our approach is more efficient and allows a fine load balancing by sharing equitably the processing of probe selection for the input group across jobs. The comparison results of our load balancing method with that used in PhylArray—for a probe design with a mean degeneracy per job equal to 37,132.25 probes—showed that our software allowed creating jobs with a small load standard deviation of only 0.5 probe while PhylGrid generated a high load standard deviation of 18,647.85 probes between jobs. The experimental results obtained have shown that the parallel implementation of our software had significantly increased performance up to 106x when running around 600 jobs on the European Computing Grid (with submission and waiting latency). The performance of our software depends on the grid resource availability and also on the number and the size of designs that can be simultaneously launched. Hence, we have to consider Grid Computing only for large designs; otherwise, the queue waiting time and the time of data transfer on and to the grid can far exceed the computing time. For small groups of sequences, the use of a computing cluster or a multiprocessor will be more efficient than the use of a grid infrastructure for latency reasons. In our case, if we do not have tens of jobs with a job running time around 12 hours, we estimate that it is not worth submitting jobs to a computing grid where our jobs may queue for hours; instead our software suggests to consider local submissions to computing clusters.

## Figures and Tables

**Table 1:** A comparison of the performance of the alignment method used in our software with that used in PhylArray [29], using 100 cores.

Aligned group	Number of sequences	Number of subgroups	Alignment time (seconds)		Speedup
			PhylArray	PhylGrid 2.0	
*Vibrio *	1,174	37	2,542	1,247	2.03
*Bacillus *	3,947	58	12,586	3,130	4.02

**Table 2:** A Comparison of our load balancing method with PhylArray [29] using 4 processors to select probes for 3 genus groups.

Group	*Citrobacter *		*Eubacterium *		*Haemophilus *	
Software	PhylArray	PhylGrid 2.0	PhylArray	PhylGrid 2.0	PhylArray	PhylGrid 2.0
Mean degeneracy	26,722.75	26,722.75	37,132.25	37,132.25	20,100.75	20,100.75
Degeneracy job 1	13,068	26,723	41,435	37,133	28,600	20,101
Degeneracy job 2	41,782	26,723	43,466	37,132	32,335	20,101
Degeneracy job 3	16,381	26,723	10,273	37,132	4,314	20,101
Degeneracy job 4	35,660	26,722	53,355	37,132	15,154	20,100
Standard deviation	**14,142.836**	**0.5**	**18,647.85**	**0.5**	**12,853.09**	**0.5**
